# Supplementary material for: The role of Kaizen in the effectiveness of Amhara metal industry and machine technology development enterprise in Ethiopia, as perceived by the employees
Source: PLoS One. 2025 May 13;20(5):e0322697. doi: 10.1371/journal.pone.0322697 (PMC12074542; doi:10.1371/journal.pone.0322697)
Supplement: Attachment — Submitted filename: Response to editor.docx [file pone.0322697.s002.docx]

1. The check list filled in and completed.
2. I ensure that I have informed both the head of the enterprise and the study participants about the what and why of this study ahead and secured their respective consent to conduct the study. This was done only with verbal communication. I don’t have a written evidence nor a recorded witness to provide as an evidence. The study involved only adult employees but not minors. Hence it didn’t require the consent of parents or guardians. Since the study did not focus on medical records of patients or archived samples, similarly, it didn’t require discussing about data anonymity nor ethics committee waiving for ensuring informed consent. (I can share the address of one or more concerned authorities of the enterprise for any one who wants to check the validity of my statements).
3. Although this study involved human participants, whether it complied with the ethical symmetry of PLOS ONE was not approved by an institutional review board (ethics committee) before the study began. That was because of two reasons: one, the study relied on survey data (filling in a questionnaire wherein participants were duly informed not to write their names nor any other symptom on the questionnaire that hints or exposes their identity for the researcher) and a few document review, data collection methods which do not expose the identity of data sources for observers; and two, there is no a review board or committee approach of protecting participant identity in the research culture of my organization.

Once you have amended this/these statement(s) in the Methods section of the manuscript, please add the same text to the “Ethics Statement” field of the submission form (via “Edit Submission”).

4. I have included the title page with the necessary information (the author or corresponding author and his affiliation).
