# Supplementary material for: The role of Kaizen in the effectiveness of Amhara metal industry and machine technology development enterprise in Ethiopia, as perceived by the employees
Source: PLoS One. 2025 May 13;20(5):e0322697. doi: 10.1371/journal.pone.0322697 (PMC12074542; doi:10.1371/journal.pone.0322697)
Supplement: Attachment — Submitted filename: REVIEWERS COMMENTS.(PONE-D-24-57548) DR. FABUNMI, ABISOLA.docx [file pone.0322697.s001.docx]

**The Role of Kaizen in the Effectiveness of Amhara Metal Industry and Machine Technology Development Enterprise in Ethiopia, as Perceived by the Staff.**

**Abstract:** The abstract effectively summarizes the study's objectives, methodology, and key findings. However:

- The abstract is well-structured but could be more concise by removing redundant phrases such as findings were noteworthy.
- The percentages reported (e.g., Kaizen accounted for approximately 44% of the enterprise’s effectiveness) are insightful but should include a note on the practical implications for practitioners and policymakers.

**Introduction:** The introduction provides an extensive background on Kaizen and its relevance. Although:

- The literature cited is comprehensive but sometimes repetitive (e.g., multiple references to the 5S principles and process-oriented nature of Kaizen).
- The research gap is articulated but could benefit from a sharper focus on the specific challenges within the Ethiopian context.
- The introduction uses strong citations, but the argument about Kaizen being superficially implemented (paper tigers) should be supported with recent evidence.

The author should streamline the discussion of Kaizen's theoretical background and strengthen the argument for the research gap with recent citations.

**Methodology:** The methodology is robust, using a mixed methods approach emphasizing quantitative techniques. However,

- The justification for the embedded mixed-methods design is adequate but could benefit from additional elaboration on why this approach was particularly suited for this study.
- Sampling techniques and validity checks are clearly outlined, but the ethical considerations section is a significant concern. The absence of institutional review board (IRB) approval is problematic, especially since the study involves human participants.

The study should provide more substantial justification for the absence of IRB approval or consider adding a retrospective approval statement from an ethical body.

Results: The results are presented and supported by appropriate statistical analyses.

- While the one-sample t-test and SEM findings are significant, the interpretation of effect sizes could be expanded. For example, what does it mean in practice that Kaizen explains 44% of organizational effectiveness?
- Visual representations (e.g., SEM diagrams) are helpful but could be better annotated to enhance comprehension.

The study should expand on the practical implications of the statistical findings and ensure that the figures are self-explanatory.

**Discussion:** The discussion is thorough and effectively connects the findings to existing literature. In that line,

- The narrative sometimes shifts to repeating results rather than interpreting them in a broader context.
- Contradictions in findings (e.g., poor Kaizen implementation but optimistic staff perceptions) are acknowledged but not fully resolved.

**Conclusion and Recommendations:** The conclusion ties the findings to actionable insights, but recommendations are broad (e.g., managers should prioritize Kaizen). Specific strategies tailored to AMIMTDE would make the recommendations more impactful. The author should offer specific, actionable recommendations grounded in the study's findings.

**Structure and Style:** The manuscript is generally well-structured, but some sections, particularly the introduction and discussion, are verbose. Also, the writing is clear but occasionally uses technical jargon that could be simplified for a broader audience. The author should reduce verbosity and ensure consistent use of terminology.

**Overall Assessment:** The manuscript is a significant contribution to understanding the role of Kaizen in organizational effectiveness within the Ethiopian context. However, several areas, particularly ethics compliance, practical recommendations, and discussion of findings, require substantial revision.

**Reviewer Recommendation:** A minor revision is required. The manuscript has potential but must address ethical concerns, refine its focus, and strengthen its discussion and recommendations to meet publication standards.
