# Supplementary material for: The role of Kaizen in the effectiveness of Amhara metal industry and machine technology development enterprise in Ethiopia, as perceived by the employees
Source: PLoS One. 2025 May 13;20(5):e0322697. doi: 10.1371/journal.pone.0322697 (PMC12074542; doi:10.1371/journal.pone.0322697)
Supplement: Attachment — Submitted filename: Response to reviewers.docx [file pone.0322697.s003.docx]

**Response to Reviewers**

| Comment category | Comments suggested by reviewers | Measures taken |
| --- | --- | --- |
| Abstract | - Remove redundant phrases or words | Removed |
|  | - Include a note on the practical implications for practitioners and policymakers about 44% of the enterprise’s effectiveness is a function of Kaizen practice | Practice, policy and research implications included |
| Introduction | - The literature cited sometimes is repetitive (e.g., multiple references to the 5S principles). | Removed repetitive references |
|  | - The research gap is articulated but could benefit from a sharper focus on the specific challenges within the Ethiopian context. | Recent literature incorporated |
|  | - The argument about Kaizen being superficially implemented (paper tigers) should be supported with recent evidence. |  |
| Methodology | - Additional elaboration on why the embedded mixed-methods design was particularly suited for this study. | Additional explanation incorporated |
|  | - The absence of institutional review board (IRB) approval | Enclosed a letter from the enterprise studied |
| Results | - The interpretation of effect sizes could be expanded | Added more explanation |
|  | - Annotate visual representations (e.g., SEM diagrams) to enhance comprehension | Added some explanatory notes |
|  | - Expanding the practical implications of the statistical findings and ensure that the figures are self-explanatory. | I tried to improve particularly for people who understand statistics |
| Discussion | - The narrative sometimes shifts to repeating results rather than interpreting them in a broader context. | Included more discussion on key points |
|  | - Contradictions in findings (e.g., poor Kaizen implementation but optimistic staff perceptions) are acknowledged but not fully resolved. | Addressed (see pp.14-15) |
| Recommendation | - Recommendations shall focus on specific strategies tailored to AMIMTDE | Made recommendations SMARTER |
| Structure and style | - Reduce verbosity and ensure consistent use of terminology | Tried to manage as much as I felt |
|  | - Simplify technical jargon for broader audience |  |
